# Effects and mechanisms of theta burst stimulation targeting individualized pre-supplementary motor area for post-stroke aphasia: study protocol for a randomized controlled trial

**DOI:** 10.3389/fneur.2026.1703554

**Published:** 2026-01-27

**Authors:** Daoran Wang, Dongdong Jiang, Chengchen Chen, Xinlei Xu, Guilan Huang, Kai Zheng, Guofu Zhang, Caili Ren

**Affiliations:** 1 Department of Rehabilitation Medicine, The Affiliated Mental Health Center of Jiangnan University, Wuxi Central Rehabilitation Hospital, Wuxi, Jiangsu, China; 2 Department of Psychiatry, The Affiliated Mental Health Center of Jiangnan University, Wuxi, Jiangsu, China

**Keywords:** aphasia, functional magnetic resonance imaging (fMRI), pre-SMA, rehabilitation, stroke, transcranial magnetic stimulation

## Abstract

**Background:**

Recent functional magnetic resonance imaging (fMRI) evidence suggests that pre-supplementary motor area (pre-SMA) activity supports language recovery in post-stroke aphasia (PSA). As a key hub within domain-general cognitive networks, the pre-SMA represents a promising target for individualized neuromodulation. While intermittent theta burst stimulation (iTBS) can enhance language recovery, its efficacy may be limited by generalized targeting strategies.

**Objective:**

This study aims to investigate the efficacy of fMRI-guided, neuronavigated iTBS targeting the individualized pre-SMA for promoting language recovery in subacute PSA and to elucidate its underlying neural mechanisms via functional connectivity (FC) analysis.

**Methods:**

In this single-center, randomized, double-blind, sham-controlled trial, 40 participants with early subacute PSA (1–3 months post-stroke) are allocated to receive either active or sham iTBS targeting the left or right pre-SMA, localized via individualized MRI mapping. Participants will undergo a 2-week intervention, with language and neuroimaging assessments conducted at baseline, immediately post-intervention, and at a 1-month follow-up. Primary outcome measures are the Western Aphasia Battery (WAB). Second outcomes measures will be including the Boston Naming Test (BNT), the Boston Diagnostic Aphasia Examination (BDAE), non-language cognitive assessment (NLCA), alongside functional connectivity analysis from resting-state fMRI.

**Expected outcomes:**

We anticipate that this trial demonstrates the efficacy of individualized pre-SMA iTBS in improving language recovery in PSA. Furthermore, we expect to identify treatment-induced neuroplastic changes in functional and structural brain connectivity. The findings could establish a novel precision neuromodulation approach for aphasia rehabilitation by identifying patient-specific biomarkers of treatment response.

**Clinical trial registration:**

https://www.chictr.org.cn/, ChiCTR2500108996.

## Introduction

Post-stroke aphasia (PSA) affects around 30% of stroke survivors and is frequently associated with enduring language impairments that significantly diminish quality of life and elevate healthcare burdens (1). These deficits spanning spontaneous speech, comprehension, repetition, and naming, not only hinder daily communication but also impair rehabilitation progress and long-term outcomes (2, 3). Speech and language therapy (SLT) remains the standard intervention for PSA, its benefits are often modest (SMD = 0.28) (1). Noninvasive brain stimulation techniques, particularly repetitive transcranial magnetic stimulation (rTMS), emerge as promising adjuncts for enhancing language recovery in PSA (2, 4, 5). The intermittent theta burst stimulation (iTBS), a specific rTMS protocol, offers unique advantages through its ability to induce an increase in cortical excitability with shorter treatment durations (6, 7).

Optimizing stimulation targets is critical for maximizing the therapeutic benefits of rTMS. Although traditional approaches have focused on perisylvian language—related areas, including inferior frontal gyrus (IFG) and superior temporal gyrus (STG) (8, 9), growing evidence highlights the importance of domain-general networks involving aphasia recovery (10, 11). Such domain-general regions underpin various cognitive processes, including working memory, reasoning, attention, and executive function (12). The cingulo-opercular network, a key domain-general network consisting of the dorsal anterior cingulate cortex, pre-supplementary motor area (pre-SMA), anterior insula, and inferior frontal gyrus, is implicated in post-stroke language recovery (12–14).

Recent neuroimaging studies reveal that the pre-SMA shows compensatory activation in patients with PSA, particularly those with left temporo-parietal and frontal infarctions (10, 15–17). This activation pattern correlates with improved performance on language tasks including picture description and naming, and serves as an independent predictor of recovery outcomes alongside traditional prognostic factors like age and aphasia severity (10, 18, 19). Functionally, the pre-SMA contributes to multiple language-related processes including speech initiation, verb generation, and auditory-motor integration (20–23). Beyond its domain-general cognitive functions, the pre-SMA is also deeply integrated within motor planning and execution circuits, forming a key interface between motor and language systems. Recent evidence suggests that language processing benefits from motor and motor-imagery training, supporting the embodied language framework (24–26). This dual role highlights that targeting pre-SMA may facilitate language recovery not only through cognitive control but also via motor-related reorganization. Nevertheless, our current protocol focuses primarily on the language-related mechanisms of pre-SMA activity, consistent with our recent finding demonstrating its association with post-stroke speech recovery (27). Anatomically, the pre-SMA's vascular supply from the bilateral anterior cerebral arteries often preserves its structural integrity in aphasia patients with left middle cerebral artery infarction (28), making it particularly suitable as a neuromodulation target in PSA patients. These converging lines of evidence strongly support the therapeutical potential of pre-SMA as a neuromodulation target for promoting language recovery after stroke. While recent work has shown that iTBS applied to the superior frontal gyrus (encompassing the pre-SMA) can improve language function in PSA patients (8), the neuroplastic mechanisms underlying these behavioral gains remain poorly understood.

Traditional rTMS approaches often employ standardized anatomical targeting based on group-level brain templates, which fails to accommodate individual variability in functional neuroanatomy and connectivity patterns. This limitation is particularly salient in prefrontal regions like the pre-SMA, where intersubject variability in both location and connectivity profiles can substantially diminish treatment efficacy (29). To tackle this challenge, we develop a personalized brain functional sector (pBFS) framework. This approach integrates resting-state functional magnetic resonance imaging (fMRI) with DTI to produce connectivity maps tailored to each individual. This method identifies precise localization of each patient's pre-SMA region based on their unique structural and functional connectivity (FC) patterns, thereby optimizing stimulation targeting (30, 31). The clinical potential of such individualized rTMS protocols has already been demonstrated in depression, schizophrenia, and neurodegenerative diseases, where connectivity-guided stimulation may serve as a promising tool for improving treatment response compared with conventional approaches (32, 33). Emerging evidence further supports the effectiveness of rTMS guided by individualized functional or structural connectivity for specific symptom management, with studies in healthy individuals further demonstrating its precision in targeting specific brain networks (34–37).

Our personalized targeting strategy aligns with the bimodal balance–recovery model of post-stroke rehabilitation (38). This framework proposes that functional recovery mechanisms are determined by the degree of structural reserve in the damaged hemisphere. When substantial white matter integrity is preserved (high structural reserve), recovery primarily occurs through remodeling of ipsilesional networks (interhemispheric competition model). Conversely, when structural damage is severe (low structural reserve), compensation predominantly relies on contralesional hemisphere recruitment (compensatory model). To operationalize this model clinically, we employ DTI to quantify the structural integrity of the left frontal aslant tract (FAT), thereby guiding individualized decisions to target either the ipsilesional or contralesional pre-SMA. Moreover, this trial specifically focuses on the early subacute phase (1–3 months post-stroke), which is recognized as a critical window for neuroplasticity and recovery. During this period, beneficial disinhibition and reorganization processes occur within motor and language networks, facilitating experience-dependent cortical remapping and responsiveness to neuromodulation (39–41). Selecting this time window is therefore intended to maximize the potential efficacy of individualized pre-SMA stimulation.

The current study has two primary objectives: (1) to determine whether fMRI-guided, individualized pre-SMA iTBS enhances language recovery in subacute PSA compared to sham stimulation, and (2) to characterize the neuroplastic mechanisms of treatment effects by examining pre-SMA-related circuit reorganization and functional connectivity changes within language network and domain-general cognitive networks. Our findings may establish a new precision medicine paradigm for aphasia rehabilitation that integrates connectivity-based targeting with biologically informed treatment protocols.

## Methods

### Trial design

**Figure 1:**
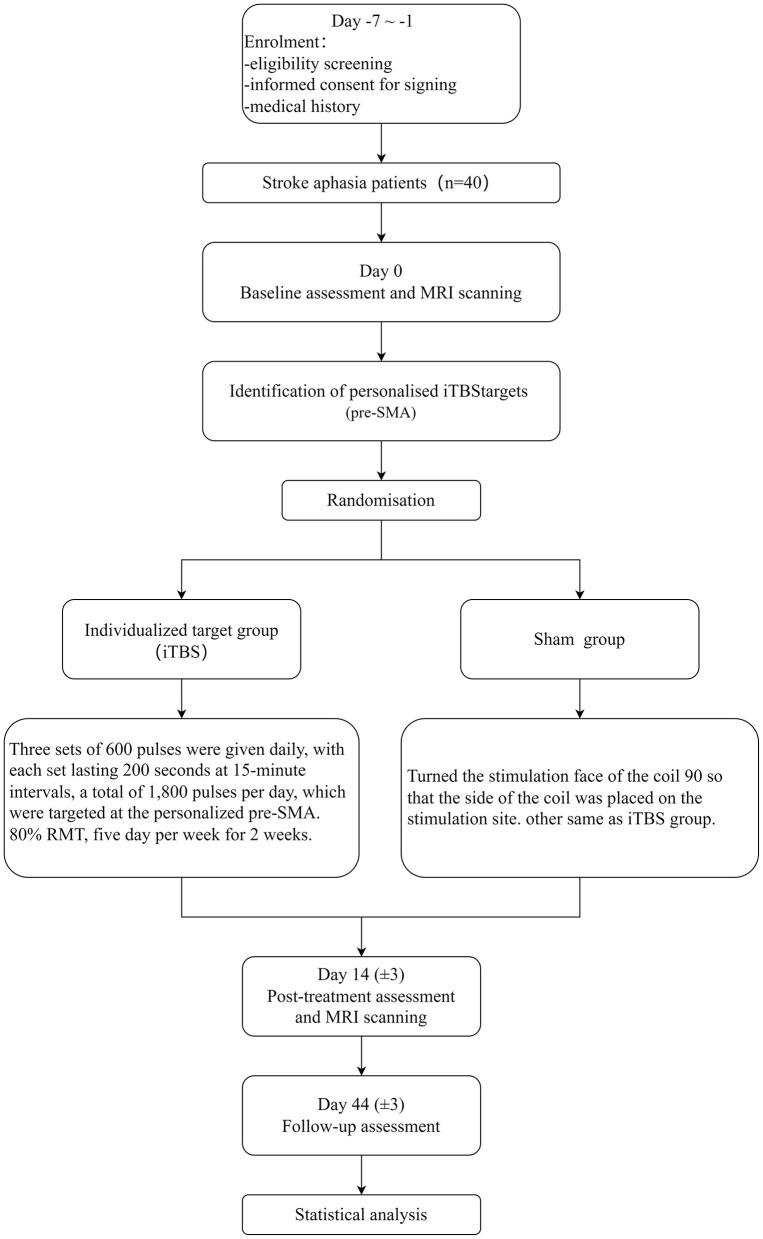
Study design and data collection.

This investigation is designed as a prospective randomized controlled trial conducted at a single center, with a double-blind, sham-controlled structure. In total, 40 individuals with subacute PSA are enrolled from Wuxi Central Rehabilitation Hospital. The ethics approval number is WXMHCIRB2025LLky116, and the clinical registry number is ChiCTR2500108996. Participants are randomly allocated at a 1:1 ratio to either the real stimulation group or the sham stimulation group. Assessments are conducted on the enrollment day (T0) and following the completion of the iTBS intervention (T1), aphasia test batteries and fMRI are performed to evaluate language functions in PSA. Figure 1 illustrates the study design and data collection procedures, while Table 1 summarizes the timeline of enrolment and assessments.

**Table 1:** Timeline of enrolment and assessments.

**Time-point**	**Screening and allocation**	**Post-randomization**		**Follow-up**
	**Day** −**7**~−**1**	**Day 0**	**Day 14 (**±**3 day)**	**Day 44 (**±**3 day)**
**Enrolment**				
Eligibility screening	√			
Informed consent	√			
Medical history	√			
Randomization	√			
MRI scanning		√	√	
**Assessment**				
NIHSS		√		
WAB		√	√	√
BDAE		√	√	√
BNT		√	√	√
NLCA		√	√	√

NIHSS, National Institute of Health Stroke Scale; WAB, Western Aphasia Battery; BDAE, Boston Diagnostic Aphasia Examination; BNT, Boston Naming Test; NLCA, non-language-based cognitive assessment.

### Participants and recruitment

Participants will be recruited from Wuxi Central Rehabilitation Hospital. Eligibility requires meeting all of the following items: (1) a confirmed diagnosis of aphasia secondary to ischaemic stroke, with symptoms lasting between 1 and 3 months; (2) left-hemisphere ischaemic stroke confirmed by brain CT or MRI scan; (3) a first-ever stroke with a single lesion; (4) Aphasia Quotient (AQ) on the WAB < 93.8; (5) aged between 50 and 75 years; (6) native Mandarin speakers with at least primary school education; (7) right-handedness; (8) intact premorbid language function; (9) informed about the procedures of iTBS treatment and brain imaging scans, and provided written informed consent.

Given the focus of the current protocol, only patients with non-fluent aphasia (as classified by the WAB) will be included. The aphasia type of all enrolled participants will be determined and documented at baseline to ensure diagnostic accuracy and sample homogeneity.

Exclusion criteria include: (1) severe auditory, visual, or cognitive deficits limiting compliance; (2) significant dysarthria (NIHSS item 10 ≥ 2); (3) impaired consciousness (NIHSS item 1a ≥ 1); (4) major psychiatric disorders such as severe depression or anxiety that may interfere with participation; (5) aphasia due to other causes (e.g., brain tumor, traumatic brain injury, Parkinson's disease, motor neuron disease); (6) history of epilepsy or substance abuse; (7) contraindications to TMS or fMRI (e.g., scalp lesions, defects of the skull, intracranial implants, pacemakers, or drug pumps).

### Sample size estimation

Sample size estimation is performed using PASS software (v15.0) with reference to prior studies (42, 43). Assuming an effect size of 0.5 for WAB-AQ, a sample size of 16 per group would provide 80% power at a two-sided significance level of 0.05. To account for an anticipated 20% dropout rate, we plan to enroll 20 participants in each group.

### Randomization

Participants will be randomly allocated in a 1:1 ratio to either the active iTBS or sham group. Randomization will be stratified by aphasia subtype (non-fluent only) to maintain balanced group allocation. The allocation sequence will be safeguarded through the use of consecutively numbered, sealed opaque envelopes, which are prepared and kept by personnel independent of the trial. Prior to the intervention, an independent researcher (not blinded) will open the corresponding envelope to determine and confirm each participant's group allocation.

### Blinding

This trial adopts a double-blind design to ensure that both participants and key study personnel remain unaware of treatment allocation. Blinding extends to participants, family members, caregivers, treating physicians, TMS operators, outcome assessors, and data analysts. Blinding procedures are outlined in the informed consent, and treatment schedules and follow-up visits are arranged to minimize potential interactions among participants.

Active and sham stimulation are delivered using a figure-of-eight double-sided coil, which has an active side for real stimulation and a placebo side that mimics the auditory and somatosensory sensations without generating an effective magnetic field. For sham stimulation, the coil is flipped, positioning the placebo side toward the scalp while maintaining an identical appearance to the active condition. To ensure full blinding, the resting motor threshold (RMT) assessment and iTBS intervention are performed by different personnel. A designated unblinded technician conducts the RMT measurement using the active side of the coil and records the motor threshold value. To preserve allocation concealment, unblinded staff label coils as “A” or “B” and perform randomization once individualized iTBS targets are determined. Only these personnel are aware of the actual mapping between labels and stimulation type. During the intervention, TMS operators are informed only which side (“A” or “B”) of the coil to use, without knowledge of the stimulation type. Imaging data are processed by blinded analysts, ensuring that group allocation remains undisclosed throughout the study.

### MRI data acquisition

All scans are conducted on a 3.0 Tesla GE SIGNA Architect scanner. High-resolution sagittal 3D T1-weighted images are acquired with a BRAVO sequence (TR/TE = 7.7/3.1 ms, FOV = 240 × 240 mm^2^, matrix = 256 × 256, slice thickness = 1.0 mm, gap = 1 mm, 176 slices). Resting-state fMRI is obtained using an EPI sequence (TR/TE = 2,500/30 ms, FOV = 240 × 240 mm^2^, matrix = 64 × 64, slice thickness = 3.5 mm, gap = 1 mm, 50 slices, 200 volumes). Participants are instructed to keep their eyes closed, stay awake, and avoid focused thinking during scanning. T2-weighted images are acquired with a turbo-spin-echo sequence (TR/TE = 5,000/120 ms, FOV = 220 × 220 mm^2^, matrix = 320 × 320, slice thickness = 5 mm, no gap, 30 slices) to identify lesion sites. Diffusion-weighted imaging (DWI) and apparent diffusion coefficient (ADC) maps are collected with TR = 3,200 ms, TE = 84 ms, and slice thickness = 5 mm. All images are visually checked for motion artifacts before analysis.

### Lesion delineation and quantification

Stroke lesions were manually delineated on T2-weighted MRI images using MRIcron (https://www.nitrc.org/projects/mricron) by two trained raters and verified by a senior neuroradiologist. Lesion masks were normalized to MNI space to calculate lesion volume and the lesion–tract overlap ratio with the left frontal aslant tract (FAT). Both indices were included as covariates in statistical analyses to control for lesion-related effects.

### pBFS mapping and white matter reconstruction

The cortical surface of a subject is subdivided, or parcellated, into functional areas based on the HCP-MMP1 (31). This atlas characterizes 180 cortical parcels on each hemisphere on the brain (360 in total) and is generated based on a large, functional neuroimaging study across a population of normal subjects in the Human Connectome Project. For each participant, the pBFS will be mapped (31) to enable correspondence between functional parcels and subject-specific white matter anatomy. Multi-direction diffusion-weighted images are preprocessed following a standardized pipeline: (1) reslicing to isotropic voxels; (2) rigid-body motion correction to the b0 reference; (3) removal of slices with excessive movement (DVARS > 2σ); (4) skull stripping using HDBET (43) with T1–DWI rigid alignment; and (5) gradient distortion correction via diffeomorphic warping between the DWI and T1 spaces (44) with T1–DWI rigid alignment. Diffusion modeling is conducted using constrained spherical deconvolution (CSD), which estimates multiple fiber orientations per voxel to resolve crossing fibers. The fiber response function is estimated per subject, and fiber orientation distribution functions (fODFs) are computed accordingly. Whole-brain deterministic tractography is performed with uniform random seeding (4 seeds per voxel; ~300,000 streamlines per brain), incorporating FA-based constraints (FA > 0.30 for seeding, termination at FA < 0.25 or curvature > 60°) to ensure anatomically plausible pathways. This reconstruction yields individualized white matter bundles connecting functional parcels across the cortex.

### Personalized stimulation target localization

**Figure 2:**
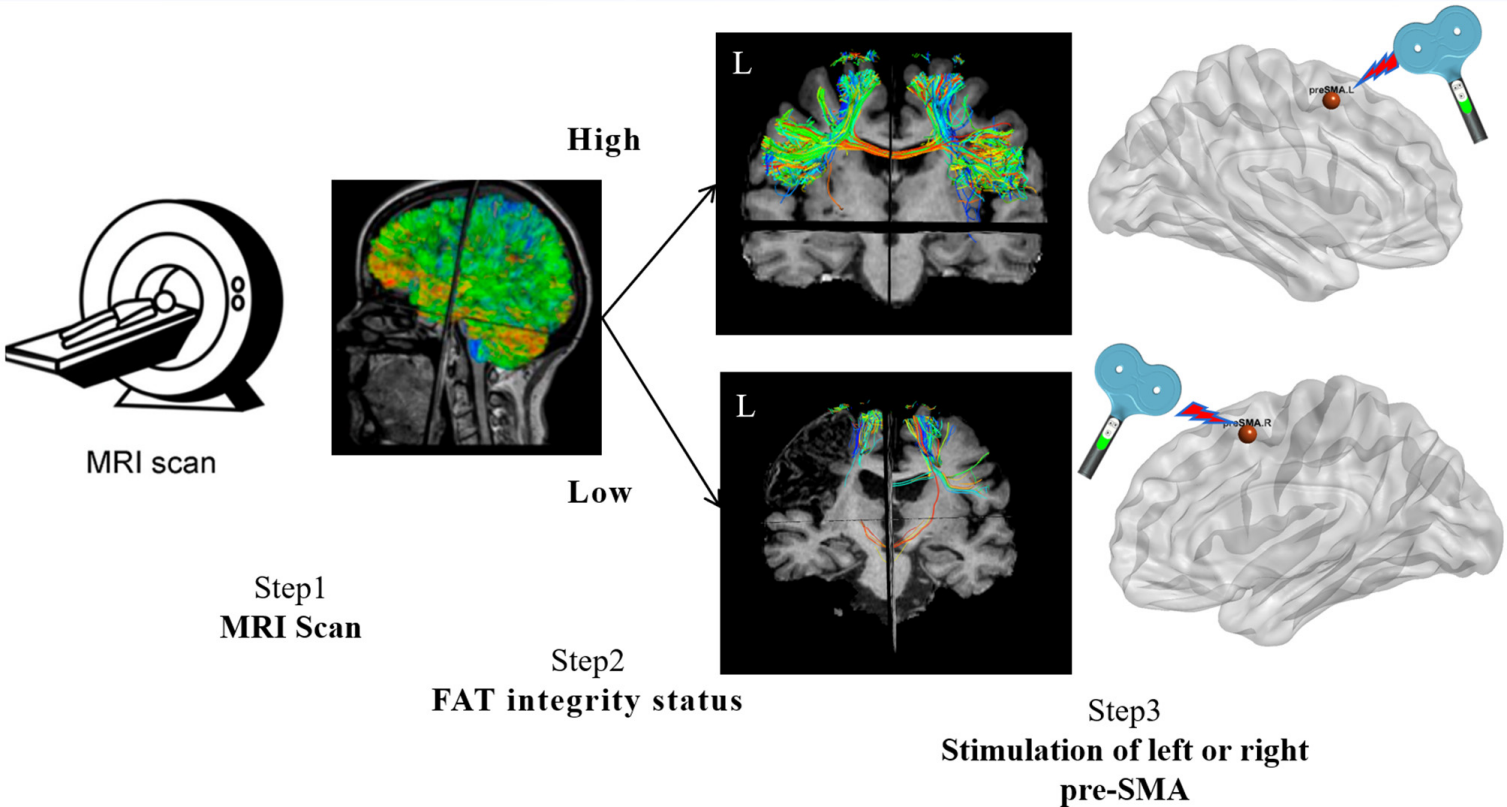
Personalised stimulation target localisation protocol. This figure illustrates the process of localising personalised TMS targets for language rehabilitation.

Before treatment commencement, two personalized stimulation targets will be identified following the steps outlined in Figure 2. The left frontal aslant tract (FAT) is reconstructed and quantitatively assessed to determine the integrity of interconnections between the left pre-SMA and the left inferior frontal gyrus (pars opercularis).

The FAT's fractional anisotropy (FA) will evaluate bilaterally. The FA ratio between the affected and unaffected sides was defined as relative FA (rFA, rFA = FAipsilesional/FAcontralesional), following the method used to evaluate the corticobulbar tract (CBT) in stroke recovery (45). As defined in prior studies, an rFA > 0.5 indicates high integrity, while an rFA ≤ 0.5 indicates low integrity. It should be noted that the specific rFA threshold for defining a functionally intact FAT has not been established in the literature. Therefore, we have extrapolated from studies on other major tracts, such as the corticospinal tract (CST) and CBT, in which an rFA > 0.5 correlates with preserved motor and swallowing function (45–47). Participants with high FAT integrity receive stimulation over the ipsilesional pre-SMA, while those with disrupted FATs are stimulated over the contralesional pre-SMA.

### Treatment

All participants will receive standard medical and rehabilitation care following established clinical guidelines (43, 48, 49). Treatments will be tailored to each individual's condition and symptoms, encompassing neuroprotective interventions, risk factor management, targeted rehabilitation exercises, and specialized nursing support.

The iTBS will be delivered via a Magneuro 100 magnetic stimulation device (Vishee Medical Technology Co., Ltd., Nanjing, China), equipped with a figure-eight stimulation coil (70 mm diameter) and integrated with a neuronavigational system (Visor 2, ANT Neuro, Netherlands), using a figure-of-eight coil capable of delivering both active and sham stimulation. The rTMS was conducted to stimulate the area over the primary motor cortex (M1) of the unaffected hemisphere (50). The RMT was defined as the minimum stimulus intensity that produced a motor evoked potential (MEP) response of at least 50 μV peak-to-peak amplitude in five out of ten trials in the abductor pollicis brevis muscle of the unaffected side (51). Following this determination, participants will undergo iTBS, delivered either as active stimulation in the experimental group or as sham stimulation in the control group. The iTBS protocol comprises three sets of 600 pulses delivered daily, with each set lasting 200 s at 15-min intervals. This results in a total of 1,800 pulses per day, which are targeted at the personalized pre-SMA. iTBS will then be delivered at 80% of the individual's rMT over the pre SMA. This intensity is based on literature demonstrating the safety and efficacy of 80% rMT stimulation applied to the superior frontal gyrus in patients with post stroke aphasia, and has been shown to induce consistent excitatory effects relevant to language recovery (8). The intervention will be delivered 5 days per week over 2 weeks, totaling 10 sessions per participant. The choice of a 15-min intersession interval was based on evidence from accelerated iTBS studies demonstrating that shorter spacing between sessions maintains safety and efficacy comparable to conventional 1-h intervals while improving clinical feasibility (52–54). All TMS operators will complete standardized training and assessment prior to the trial to ensure consistency. To ensure procedural consistency, each participant will receive all treatments from a single assigned TMS operator. Following iTBS treatment, a certified speech-language therapist will administer a 30-min session of SLT for each participant.

### Outcome measures

The primary outcome of this study is the WAB, which includes four subtests: spontaneous speech, auditory verbal comprehension, repetition, and naming. Maximum attainable scores are 20 for spontaneous speech, 200 for auditory comprehension, and 100 each for repetition and naming. The AQ is then computed as: (spontaneous speech + auditory comprehension/20 + repetition/10 + naming/10) × 2. The AQ provides an index of aphasia severity and is used to evaluate changes in language function, with a maximum score of 100, a normal range of 98.4–99.6, and scores below 93.8 indicating aphasia.

Secondary outcome measures include BNT, NLCA, and resting-state fMRI. The BNT assesses confrontation naming using 30 object pictures. The NLCA evaluates nonverbal cognitive function across five domains—visuospatial ability, attention, memory, logical reasoning, and executive function—using visual materials, with a total score of 80; scores ≥75 are considered normal.

## Data analysis

### Behavioral data

Repeated-measures ANOVAs are used to assess changes in WAB, BNT, NLCA scores, and speech parameters, with phase (pre- vs. post-treatment) as a within-subject factor and group (active vs. sham iTBS) as a between-subject factor. *Post hoc* comparisons are adjusted using the Bonferroni method. Data normality is checked with the Kolmogorov–Smirnov test; non-normally distributed variables are analyzed using the Wilcoxon signed-rank test. An intention-to-treat (ITT) approach is applied in the presence of missing data.

In cases where the assumptions of normality or homogeneity of variances are violated, a non-parametric Aligned Rank Transform (ART) ANOVA was applied to test for main and interaction effects. This approach preserves the interpretability of ANOVA-type models while providing robustness to deviations from normality. For *post-hoc* comparisons, the Wilcoxon signed-rank test (within-group) and Mann–Whitney *U*-test (between-group) are used with Bonferroni correction.

To further explore variability in treatment response, a sub-analysis will be performed within the non-fluent aphasia cohort, stratifying participants according to their baseline WAB-AQ scores to examine severity-related differences in iTBS efficacy. Participants will be categorized into mild (WAB-AQ: 50.4–93.7), moderate (30.1–50.3), and severe (0–30) aphasia groups based on their baseline scores (55). Changes in primary behavioral outcomes (WAB, BNT, NLCA) will be compared across these severity strata using mixed-design ANOVAs, followed by Bonferroni-corrected *post hoc* tests. This analysis aims to explore potential differential treatment responses across varying degrees of language impairment.

### fMRI preprocessing and functional connectivity analysis

The resting-state fMRI data are preprocessed using Statistical Parametric Mapping (SPM8; http://www.fil.ion.ucl.ac.uk/spm) and the Data Processing Assistant for Resting-State fMRI [DPARSF; (56)]. Preprocessing of the fMRI data involved the following steps: (1) discarding the first 10 volumes to allow signal stabilization and participant adaptation, leaving 190 images for analysis; (2) correcting for slice timing and head motion, with all participants exhibiting less than 2 mm displacement and 2° rotation; (3) co-registering T1-weighted images to the mean functional image and segmenting into gray matter, white matter, and cerebrospinal fluid; (4) regressing out confounding signals, including motion parameters and BOLD signals from ventricles and white matter; (5) normalizing functional images to MNI space using segmentation parameters (57), and resampling to 3-mm isotropic voxels; (6) smoothing with a 6-mm FWHM Gaussian kernel; and (7) applying temporal band-pass filtering (0.01–0.08 Hz) to remove low-frequency drift and high-frequency noise.

After the preprocessing, functional connectivity (FC) maps are calculated by computing Pearson correlation coefficients between the mean time series of each seed and all brain voxels, followed by conversion to Fisher *z*-values. Seed regions are defined as 6-mm-radius spheres centered at the ipsilesional pre-SMA (*x* = −4, *y* = 8, *z* = 50) and contralesional pre-SMA (*x* = 4, *y* = 8, *z* = 50), based on previous studies (10).

FC changes are analyzed at the voxel level using a 2 × 2 mixed-design repeated-measures ANOVA, with group (active vs. sham) as a between-subject factor and time (pre- vs. post-treatment) as a within-subject factor. Voxel-wise multiple comparisons are corrected using the false discovery rate (FDR, *p* < 0.05, cluster-level). *Post hoc* analyses further examine FC alterations within the pre-SMA–language network. Correlation analyses between FC changes and behavioral improvements are performed using Pearson correlation coefficients, with *p*-values FDR-corrected for multiple comparisons (*p* < 0.05).

### Diffusion data analysis

All diffusion data are preprocessed offline using FSL 5.0.9 (https://fsl.fmrib.ox.ac.uk/fsl/fslwiki/FSL) (58). Preprocessing included correction for subject motion and eddy currents, with the b-matrix adjusted to account for rotational motion. Diffusion tensors are then estimated using nonlinear least squares fitting. Whole-brain tractography is conducted with a 2-mm uniform seed point resolution, applying termination criteria of FA < 0.2, angle < 30°, and fiber length < 50 mm.

The mean fractional anisotropy (FA) of the FAT is extracted to evaluate its microstructural integrity. FA reflects the directional dependency of water diffusion within fiber tracts, with values ranging between 0 (representing isotropic diffusion) and 1 (representing diffusion along a single direction), with higher values indicating greater fiber coherence and organization. FA is considered sensitive to microstructural properties such as myelination and axonal density, making it a useful marker for detecting subtle changes in white matter (59). FA values of the FAT are analyzed using a two-way mixed-design repeated-measures ANOVA, with group (active vs. sham) as a between-subject factor and time (pre- vs. post-treatment) as a within-subject factor. *Post hoc* pairwise comparisons receive Bonferroni correction. To assess structure–function–behavior relationships, correlations are computed between FA changes, FC alterations, and behavioral improvements using Pearson correlation analyses, with *p*-values FDR-corrected for multiple comparisons (*p* < 0.05).

### Safety

Adverse effects are defined as any negative experiences experienced by participants during iTBS or MRI procedures. Investigators will record all adverse events occurring during treatment and up to 1 week afterward. Seizures, the most severe TMS-related adverse events with an estimated risk of 0.02%, are expected only during or immediately following iTBS. MRI-related side effects will be reported within 24 hours, with ear protection provided to limit acoustic disturbance. Any serious incidents will be promptly reported to the Medical Research Ethics Committee of Wuxi Central Rehabilitation Hospital.

## Discussion

This randomized controlled clinical trial with a sham intervention aims to explore the neural and behavioral effects of personalized iTBS targeting the ipsilesional or contralesional pre-SMA. We hypothesize that stimulating the personalized pre-SMA with iTBS can enhance the effects of SLT on language recovery in PSA by modulating pre-SMA functional connectivity and inducing microstructural changes in the FAT, both of which are involved in language processing. The findings may offer evidence supporting the pre-SMA as a potential optimal stimulation target for treating subacute PSA. This study is designed to ascertain the integrity of the lesion-side FAT guided by DTI, and to carry out personalized intervention based on this. It is found that personalized pre-SMA iTBS stimulation might be a new neuromodulation approach for PSA recovery, as it is associated with the recovery of aphasia. Several studies indicate that rTMS has emerged as a potential therapeutic tool for ameliorating language dysfunction associated with PSA, yet the heterogeneity of treatment outcomes remains relatively high, which is attributed to variations in therapeutic targeting (60, 61). An extensive body of evidence indicates that the pre-SMA, as a specific target, is linked to the recovery process in patients with PSA, which further implies that it may serve as a potential stimulation target for cranial magnetic stimulation CMS interventions in PSA treatment (10). The development of fMRI-guided TMS-based personalized targeting for research may improve the therapeutic efficacy and therapeutic response rate of neuromodulation techniques, become an emerging means of stroke treatment, and be able to effectively reduce the burden of stroke patients' families and save social resources (62).

The correlation between the increased efficacy of fMRI-based individualized targeting in the recovery of speech function in PSA and the mechanism of neural remodeling has not yet been explored: Prior studies document that the pre-SMA serves as a key node in the reorganization of the neural speech network and neural cognitive network in patients with PSA (63), and our preliminary experiments confirm the important role of the pre-SMA in the recovery of speech function in patients with PSA, as demonstrated by the resting-state fMRI-functional connectivity analyses. which exerts a crucial role in the process of speech function recovery, laying a scientific foundation for the clinical research of this project. This project is to explore the efficacy of fMRI-based individualized targeting in enhancing the language recovery for PSA, and then to provide a basis for decision-making on individualized TMS treatments for PSA.

## Limitation

The current study presents several limitations that warrant consideration. First, the recruitment of participants with PSA remains challenging, resulting in relatively small sample sizes in many studies. This limitation compromises statistical power and consequently restricts the reliability and generalizability of research findings. Future studies with larger cohorts will incorporate aphasia subtype-based stratification to better capture potential differential responses across aphasia phenotypes. Furthermore, the absence of long-term follow-up in this investigation confines our observations to short-term therapeutic outcomes, thereby hindering a comprehensive understanding of treatment efficacy in sustained recovery processes. This methodological gap may obscure the longitudinal trajectory of therapeutic interventions.

Additionally, substantial heterogeneity exists within the PSA population regarding lesion topography, infarct volume, aphasia subtypes (e.g., Broca's aphasia, Wernicke's aphasia), and severity stratification (64). Such clinical variability complicates comparative effectiveness analyses across studies, as differential treatment responses among patients may substantially influence outcome interpretation. The inherent neurobiological diversity in this population necessitates cautious generalization of therapeutic outcomes. Perfusion imaging such as ASL was not included in the current protocol. Future studies should integrate cerebral blood flow measurements to better capture hemodynamic contributions to language recovery.

Lastly, although the pre-SMA also participates in motor control and planning, this protocol prioritizes language-related outcome measures to specifically evaluate speech network modulation. Including motor assessments such as the Fugl–Meyer or ARAT will be considered in subsequent studies to further disentangle motor- and language-specific contributions to recovery mechanisms.
